# Supplementary material for: In-host evolution of Staphylococcus epidermidis in a pacemaker-associated endocarditis resulting in increased antibiotic tolerance
Source: Nat Commun. 2019 Mar 8;10:1149. doi: 10.1038/s41467-019-09053-9 (PMC6408453; doi:10.1038/s41467-019-09053-9)
Supplement: Supplementary file 1 — Supplementary Information [file 41467_2019_9053_MOESM1_ESM.pdf]

Supplementary material for:

In-host evolution of *Staphylococcus epidermidis* in a pacemaker-associated endocarditis resulting in increased antibiotic tolerance

Dengler Haunreiter et al.

Content

Figures

1) Pulsed-field-gel-electrophoresis of the clinical isolates
2) Maximum-likelihood tree
3) RpoB gene sequences
4) Biofilms treated with DNase and proteinase K
5) Cultivation time to reach OD 0.1 and minimum doubling times
6) Logarithmic plots of the time-kill curve data
7) Antibiotic clearance of bacteria in biofilms
8) Time to single-cell first division in DMEM

Tables

1) Minimum inhibitory concentrations (MICs)
2) Overview of the reference strains used for tree construction
3) Summary of the variants found

Supplementary Figure 1: Pulsed-field gel electrophoresis (PFGE) of *S. epidermidis* isolates obtained from the patient.

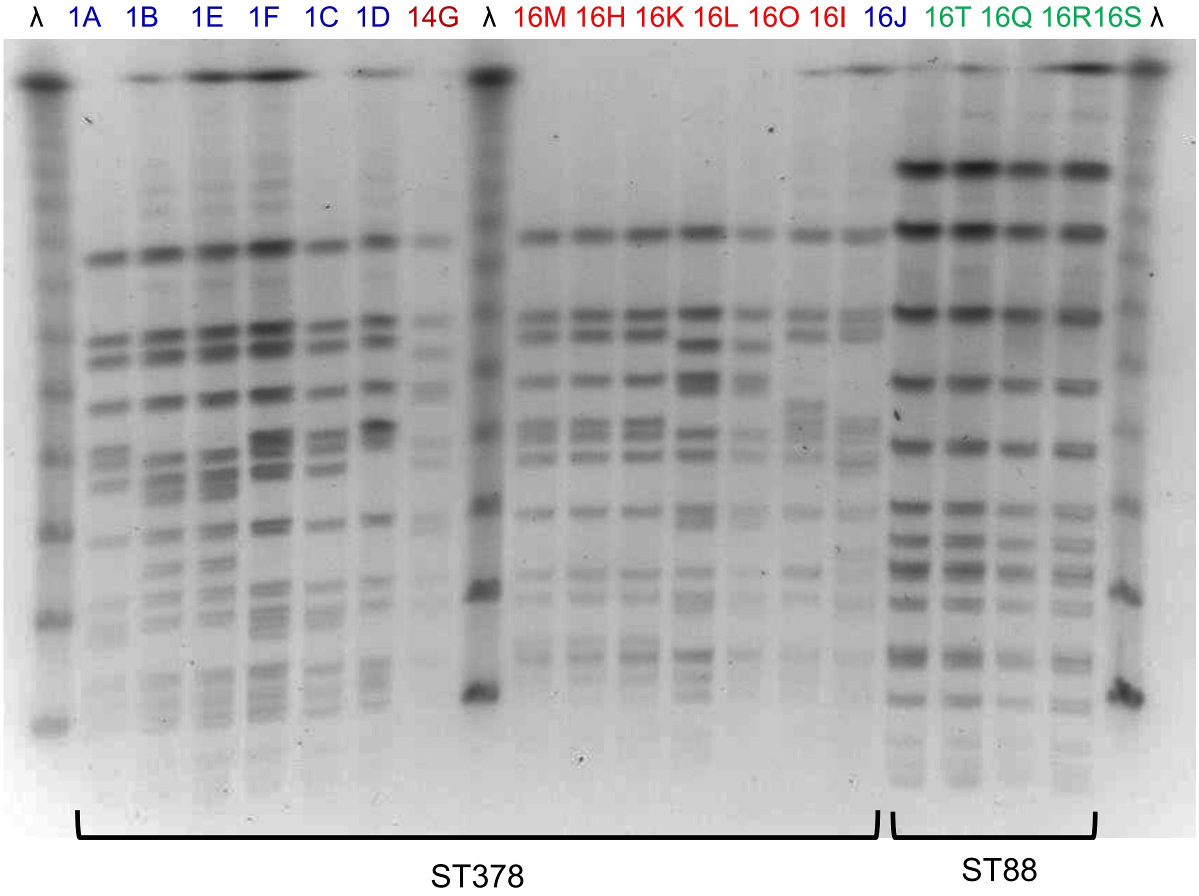

DNA was extracted from *S. epidermidis* isolates and digested with the restriction enzyme SmaI**.** Blue and red labelling of the isolate numbers indicate the ST378 sub-groups I and II, respectively, green labelling indicates ST88 isolates.

Supplementary Figure 2: Maximum likelihood tree of the ST378 isolates.

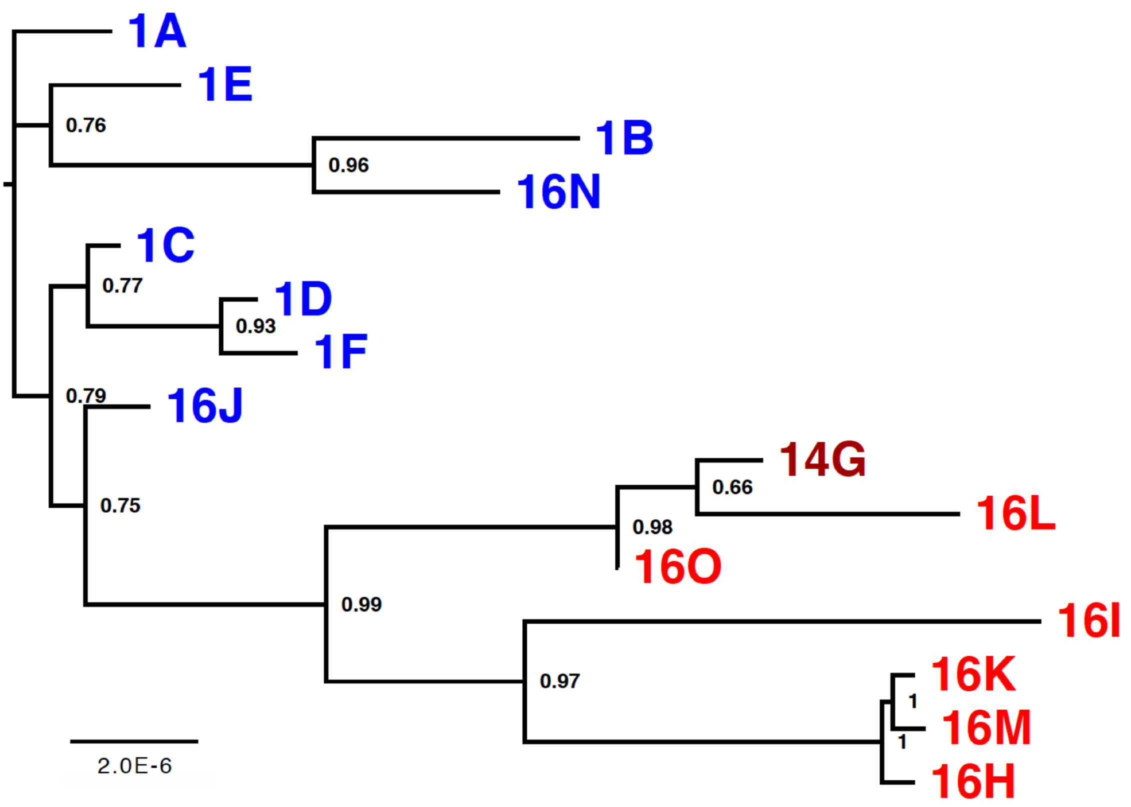

The tree was built on the concatenated core gene alignment. The values displayed on the nodes are local support values computed with the Shimodaira-Hasegawa test by FastTree and the scale bar corresponds to $2x10^{-6}$ SNPs per site per year. Label colouring of the isolate number highlights the two sub-groups: group I blue and group II red. The darker red indicates the clinical isolate retrieved from the blood culture.

Supplementary Figure 3: Non-synonymous mutations in the *rpoB* gene.

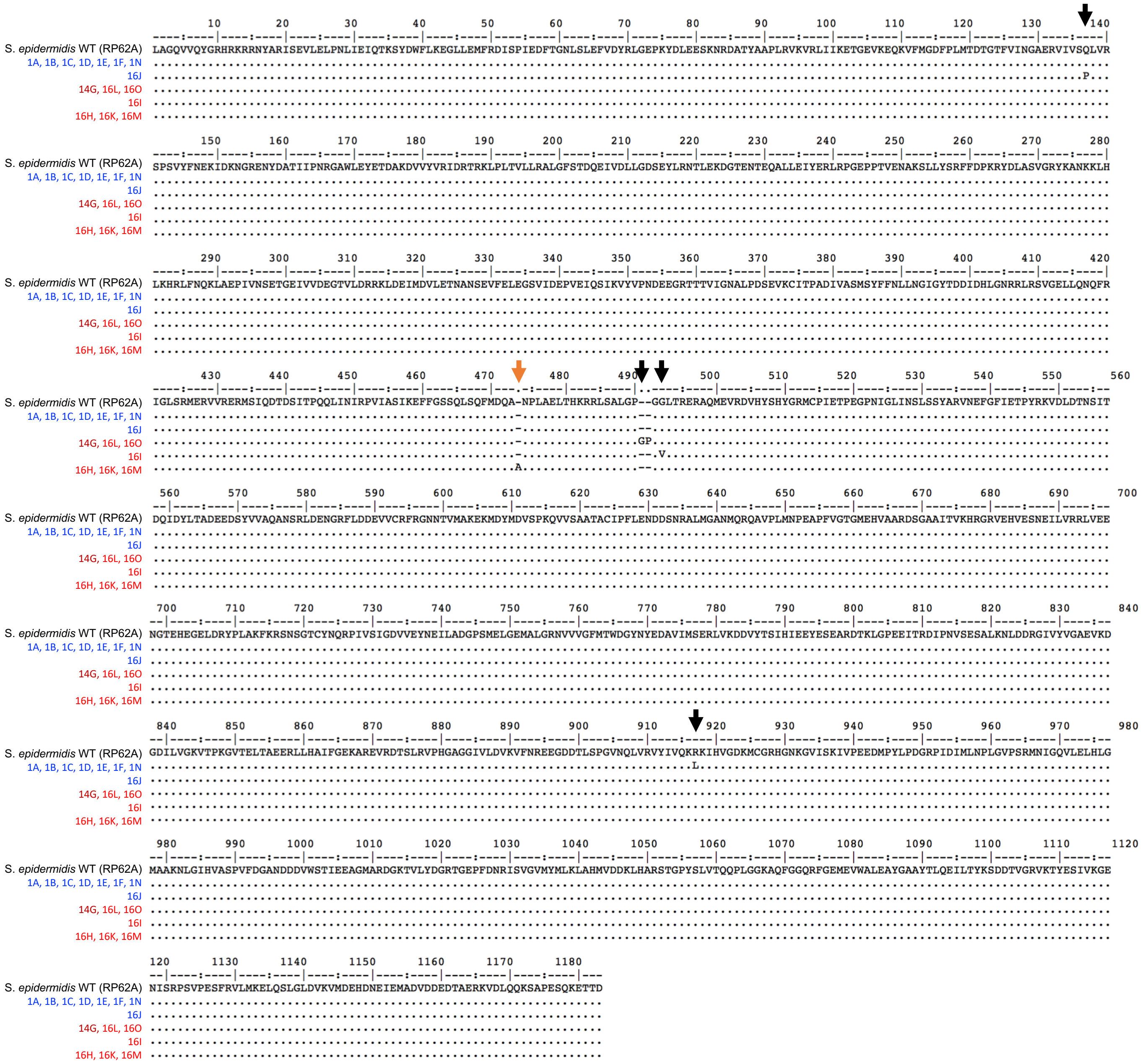

Multiple alignment of the ST378 isolates RpoB to the reference RP62A RpoB amino acid sequences (Genbank accession: AAW53580).

Dots indicate matches with the reference. Five non-synonymous mutations (2 insertions and 3 SNPs) were found in different *S. epidermidis* clinical isolates and are indicated by arrows. The orange arrow points at the mutation found in the isolates 16H, 16K and 16M, which displayed resistance to rifampicin (Ala473_Asn474insAla).

Supplementary Figure 4: Biofilm treatment with Proteinase K and DNase I of group II isolates.

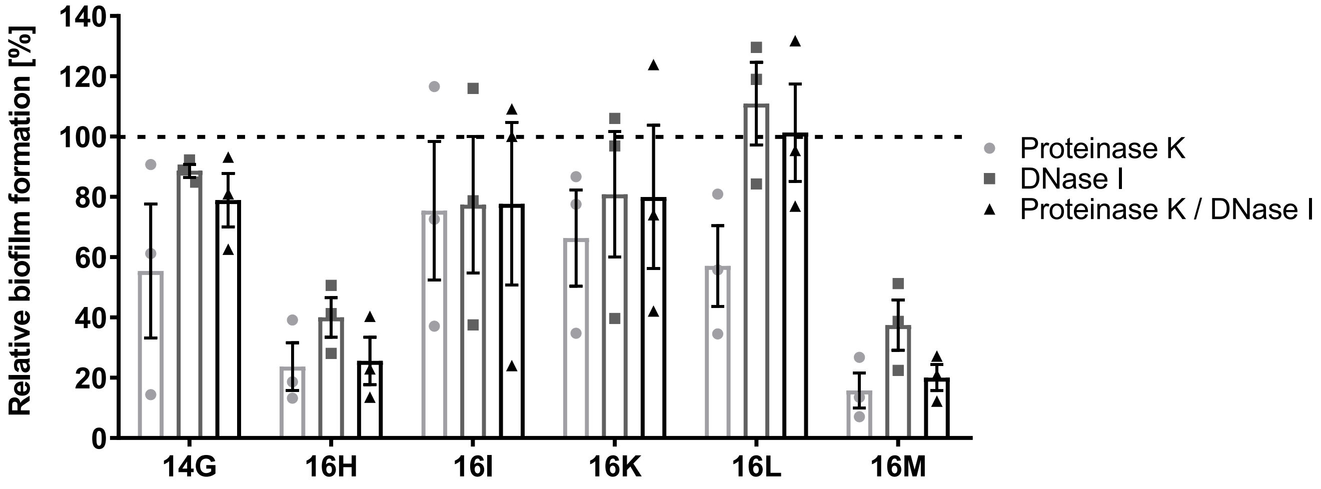

Biofilms were grown in presence of DNase I, proteinase K or both and biofilm formation was quantified after 24 hours by crystal violet staining. Relative biofilm formation compared to untreated biofilm is shown. Averages with standard deviation of three replicates are shown.

Supplementary Figure 5: Cultivation time to reach OD 0.1 and minimal doubling times of *S. epidermidis* isolates.

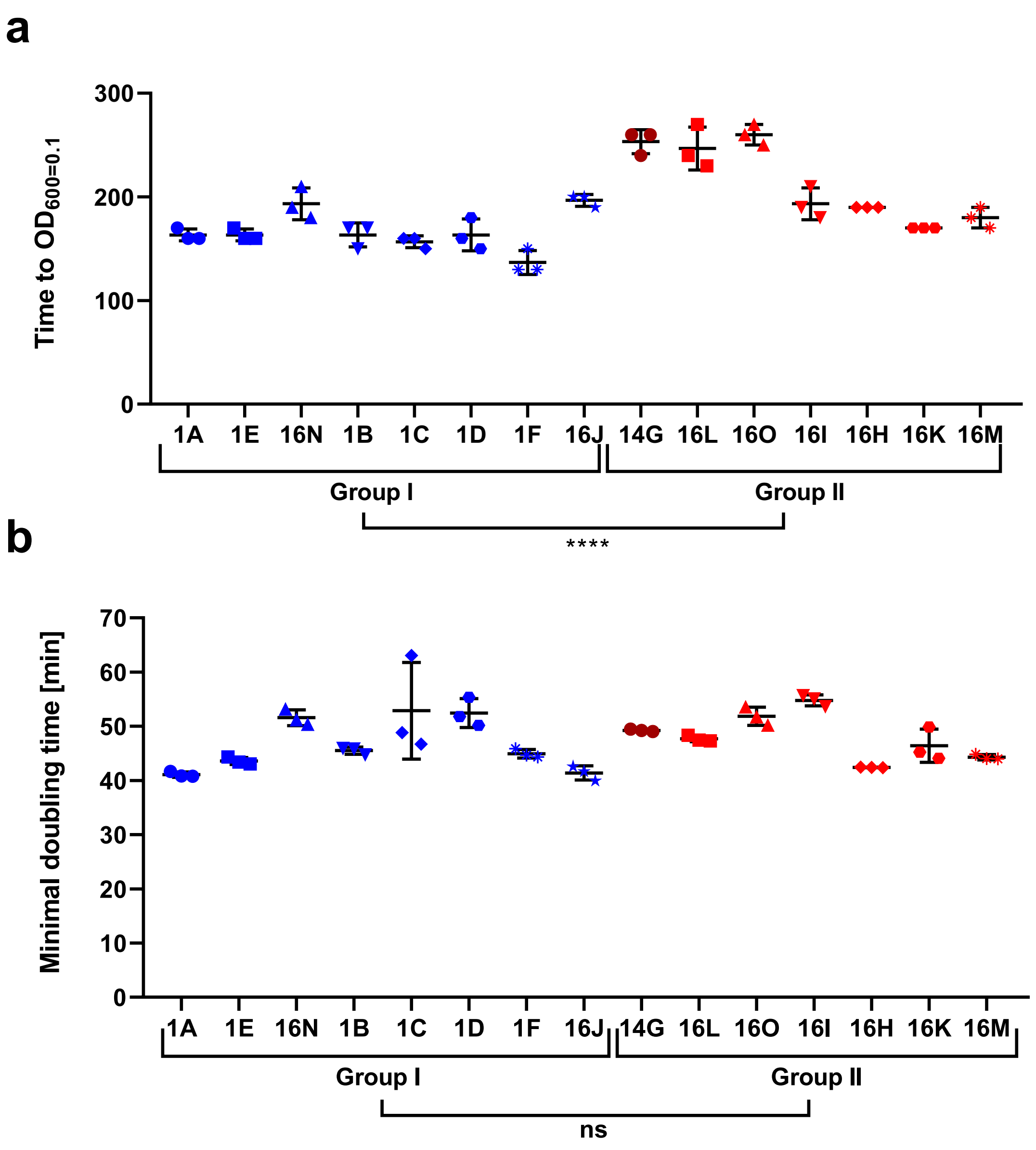

The data was extracted from the growth curves shown in Figure 4a.

a Time to reach an OD600 of 0.1. Statistical significance between the two groups was determined by Welch two-sample t-tests (See figure legend 4a) **b** Minimal doubling times, calculated with an one hour interval from growth curves in TSB in a 96-well plate. Statistical significance between the two groups was determined by Welch two-sample t-tests (N=46, t(42.397)=-0,98, P=0.33). Averages with standard deviation of three replicates are shown.
**** p<0.0001; ns, not significant.

Supplementary Figure S6: Alternative visualization of the time-kill curve experiments.

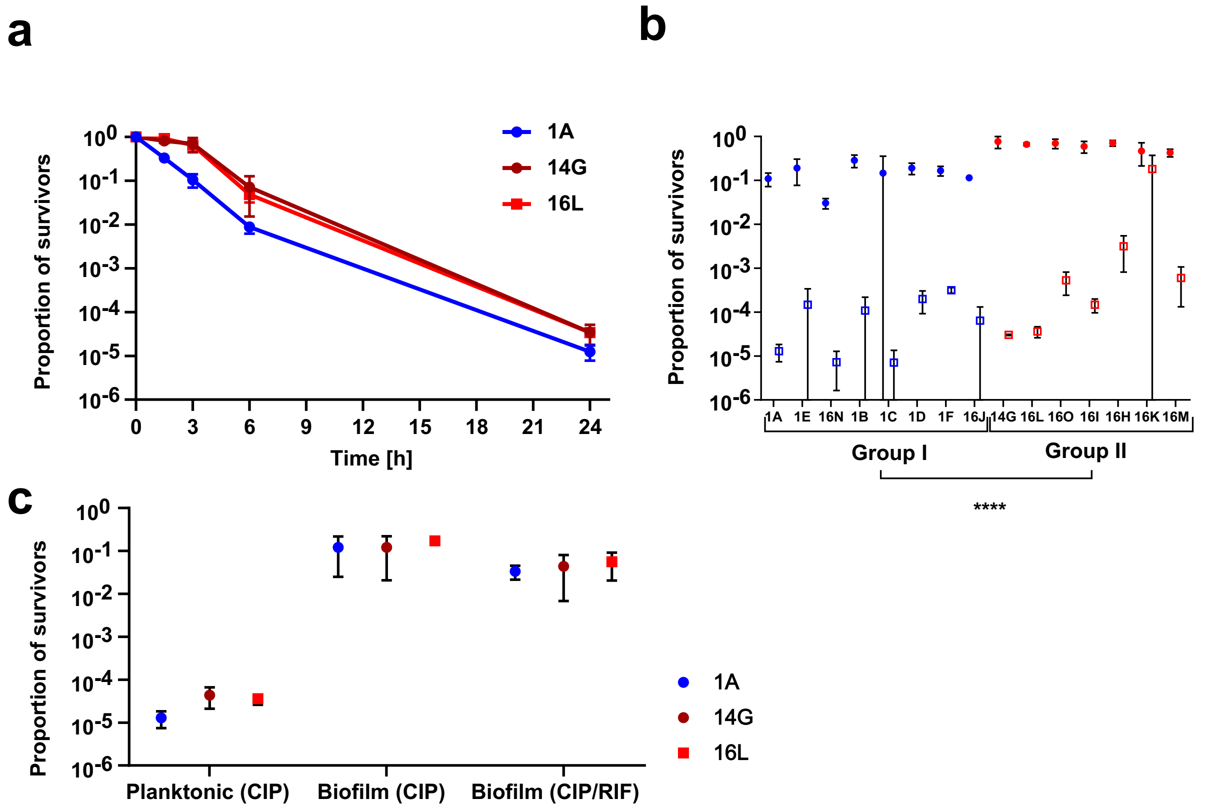

Alternative plotting with logarithmic axis of, **a** Figure 6B, **b** Figure 6C and **c** Figure 6E. Averages with standard deviation of 3 replicates are shown. Statistical significance of survival to 3h antibiotic exposure between the two groups was determined by Welch two-sample t-tests (See figure legend 6c). ****, $p<0.0001$

Supplementary Figure 7: Antibiotic clearance of bacteria in biofilms.

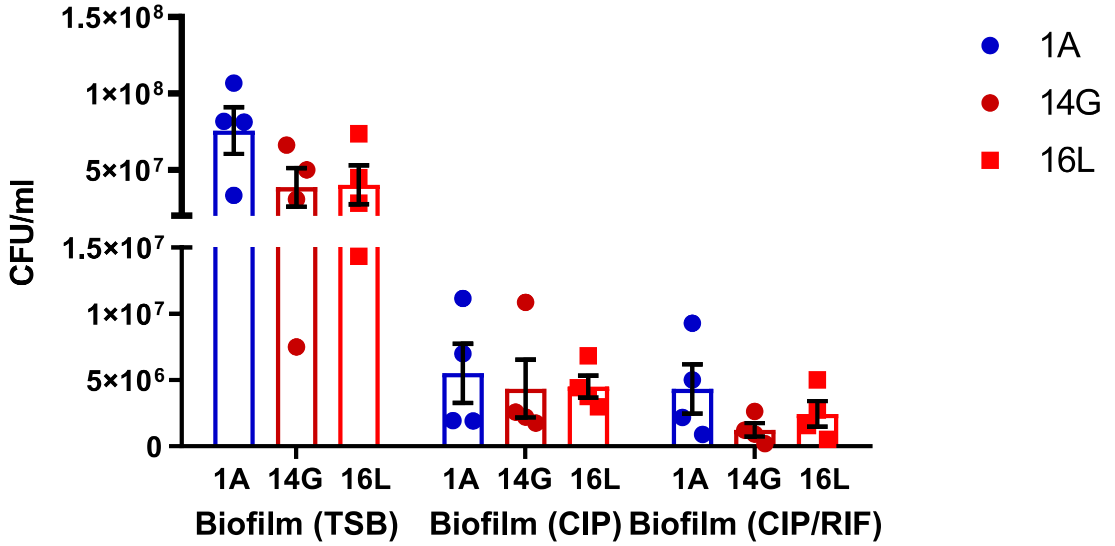

Antibiotic killing of bacteria embedded within a biofilm by ciprofloxacin and a combination of ciprofloxacin and rifampicin as compared to untreated biofilm. The amount of viable bacteria was quantified by assessing the number of CFUs grown on agar plates. Averages with standard deviation of three replicates are shown.

Supplementary Figure 8: Time to single cells' first division

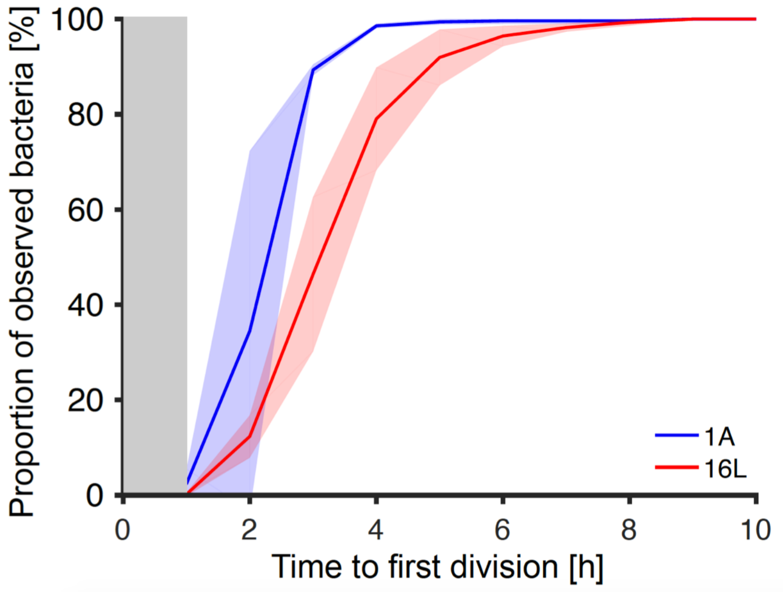

Curves show averages of three replicates and shaded areas depict standard deviation. Experiment performed in DMEM 4.5g/l glucose with 10% FBS. The time for 80% of the population to resumed growth differed by 1.5 hours between isolate 1A (3.3 ± 0.3 h) and 16L (4.8 ± 0.6 h). The grey zone marks the period at the beginning of the experiment where cell divisions could occur but not be observed.

Supplementary Table 1: Minimum Inhibitory concentrations (MIC) of the ST378 clinical isolates

Isolate number*	MIC [mg/L]		
	Cirpofloxacin	Rifampicin	Erythromycin
1A	0.062 - 0.25 (S)	< 0.0078 (S)	64 - 128 (R)
1B	0.125 - 0.5 (S)	< 0.0078 (S)	32 (R)
1C	0.062 - 0.25 (S)	< 0.0078 (S)	64 - 128 (R)
1D	0.125 - 0.25 (S)	< 0.0078 (S)	64 - 128 (R)
1E	0.062 - 0.5 (S)	< 0.0078 (S)	32 - 64 (R)
1F	0.125 - 0.25 (S)	< 0.0078 (S)	64 - 128 (R)
16J	0.062 - 0.125 (S)	0.015 - 0.031 (S)	64 (R)
16N	0.062 (S)	0.0078-0.031 (S)	16 - 32 (R)
14G	0.062 - 0.125 (S)	0.0078 - 0.015 (S)	16 - 32 (R)
16H	0.125 - 0.5 (S)	1 - 2 (R)	16 - 32 (R)
16I	0.062 - 0.25 (S)	< 0.0078 (S)	8 - 16 (R)
16K	0.125 - 0.5 (S)	1 - 2 (R)	32 - 64 (R)
16L	0.125 - 0.25 (S)	0.015 (S)	16 - 32 (R)
16M	0.125 - 0.5 (S)	1 - 2 (R)	32 - 64 (R)
16O	0.25 (S)	0.125 (S)	8 - 32 (R)

Blue and red labelling of the isolate numbers indicate the ST378 sub-groups I and II, respectively. The darker red indicates the clinical isolate retrieved from the blood culture.
Resistant (R), susceptible (S).

Supplementary Table 2: Reference genomes used for tree construction

Organism/Name	Strain name	MLST[1]	MLST reference	arCC	aroE	gtr	mutS	pyrR	tpiA	yqiL	BioSample	BioProject	Assembly	Level[2]	Size (Mb)	GC%	Replicons	Gene	Protein	Release Date	Modify Date
Staphylococcus epidermidis ATCC 12228	ATCC 12228	ST8	Conlan *et al.* 2012	2	1	7	1	1	1	1	SAMN02603126	PRJNA279	GCA_000007645.1	CG	2.56461	32.05	chromosome:NC_004461.1/AE015929.1<br>plasmid pSE-12228-01:NC_005008.1/AE015930.1<br>plasmid pSE-12228-02:NC_005007.1/AE015931.1<br>plasmid pSE-12228-03:NC_005006.1/AE015932.1<br>plasmid pSE-12228-04:NC_005005.1/AE015933.1<br>plasmid pSE-12228-05:NC_005004.1/AE015934.1<br>plasmid pSE-12228-06:NC_005003.1/AE015935.1	2558	2482	20.12.02	03.08.16
Staphylococcus epidermidis RP62A	RP62A	ST10	Conlan *et al.* 2012	1	1	1	1	3	1	1	SAMN02603975	PRJNA64	GCA_000011925.1	CG	2.64384	32.2	chromosome:NC_002976.3/CP000029.1<br>plasmid pSERP:NC_006663.1/CP000028.1	2625	2462	09.01.02	22.03.17
Staphylococcus epidermidis PM221	PM221	ST184	Savijoki *et al.* 2014	16	1	2	1	2	1	1	SAMEA3139103	PRJEB5186	GCA_000751035.1	CG	2.59751	31.93	chromosome I:NZ_HG813242.1/HG813242.1<br>plasmid II:NZ_HG813243.1/HG813243.1<br>plasmid III:NZ_HG813244.1/HG813244.1<br>plasmid IV:NZ_HG813245.1/HG813245.1<br>plasmid V:NZ_HG813246.1/HG813246.1	2589	2399	14.08.14	24.03.17
Staphylococcus epidermidis SEI	SEI	unknown (nearest 722, 436, 387)	CGE	1	13	7	6	2	5	33	SAMN02928813	PRJNA244351	GCA_000759555.1	CG	2.53831	32.03	chromosome:NZ_CP009046.1/CP009046.1<br>plasmid unnamed:NZ_CP009047.1/CP009047.1	2504	2311	29.09.14	24.03.17
Staphylococcus epidermidis 14.1.R1	14.1.R1	unknown (nearest 573)	CGE	49	29	9	30	7	5	56	SAMN06167133	PRJNA358169	GCA_001956655.2	CG	2.62533	32.18	chromosome:NZ_CP018842.1/CP018842.1<br>CP018841.1:CP018841.1<br>plasmid pHOB1:NZ_CP018843.1/CP018843.1<br>plasmid pHOB2:NZ_CP018844.1/CP018844.1	2628	2378	05.01.17	05.02.18
Staphylococcus epidermidis 1457	1457	ST86	CGE	1	2	2	1	1	1	1	SAMN02640609	PRJNA377759	GCA_002085695.1	CG	2.47007	32.27	chromosome:NZ_CP020463.1/CP020463.1	2433	2283	11.04.17	30.01.18
Staphylococcus epidermidis DAR1907	DAR1907	ST2	CGE	7	1	2	2	4	1	1	SAMN07444319	PRJNA308322	GCA_002850315.1	CG	2.72755	32.1	chromosome:NZ_CP013943.1/CP013943.1	2748	2584	03.01.18	11.01.18
Staphylococcus epidermidis FDAARGOS_153	FDAARGOS_153	ST5	CGE	1	1	1	2	2	1	1	SAMN03996299	PRJNA231221	GCA_002944995.1	CG	2.55197	32.1	chromosome:NZ_CP014119.1/CP014119.1<br>plasmid unnamed1:NZ_CP014118.1/CP014118.1<br>plasmid unnamed2:NZ_CP014120.1/CP014120.1<br>plasmid unnamed3:NZ_CP014117.1/CP014117.1<br>plasmid unnamed4:NZ_CP014116.1/CP014116.1	2498	2331	08.02.18	02.03.18
Staphylococcus epidermidis FDAARGOS_161	FDAARGOS_161	ST20	CGE	1	1	2	2	1	1	3	SAMN03996306	PRJNA231221	GCA_002954055.1	CG	2.51573	32.18	chromosome:NZ_CP014132.1/CP014132.1<br>plasmid unnamed1:NZ_CP014130.1/CP014130.1<br>plasmid unnamed2:NZ_CP014131.1/CP014131.1	2466	2304	20.02.18	04.03.18
Staphylococcus epidermidis BPH0662	BPH0662	ST2	Lee *et al.* 2016	7	1	2	2	4	1	1	SAMEA3982825	PRJEB13975	GCA_900086615.1	CG	2.84117	31.97	chromosome 1:NZ_LT571449.1/LT571449.1<br>plasmid 2:NZ_LT571450.1/LT571450.1<br>plasmid 3:NZ_LT571451.1/LT571451.1	2855	2699	12.05.16	28.03.17
Staphylococcus epidermidis SE90	SE90	unknown (nearest 442)	CGE	28	22	13	4	3	4	56	SAMN07702384	PRJNA412200	GCA_002749455.1	Ch	2.4186	32.04	chromosome:NZ_CP024408.1/CP024408.1<br>plasmid p1_90:NZ_CP024409.1/CP024409.1<br>plasmid p2_90:NZ_CP024410.1/CP024410.1	2383	2225	03.11.17	06.11.17
Staphylococcus epidermidis SE95	SE95	unknown (nearest 743)	CGE	65	3	5	5	3	22	45	SAMN07702411	PRJNA412208	GCA_002749515.1	Ch	2.43784	32.07	chromosome:NZ_CP024437.1/CP024437.1<br>plasmid p_1_95:NZ_CP024438.1/CP024438.1<br>plasmid p_2_95:NZ_CP024439.1/CP024439.1<br>plasmid p_3_95:NZ_CP024440.1/CP024440.1<br>plasmid p_4_95:NZ_CP024441.1/CP024441.1	2399	2216	03.11.17	06.11.17
Staphylococcus epidermidis GTH12	GTH12	unknown (nearest 454, 115, 89, 88)	CGE	1	1	2	1	2	1	45	SAMN08555375	PRJNA434386	GCA_003119275.1	Ch	2.49953	33.2	chromosome:CP028282.1	-	-	11.05.18	11.05.18
Staphylococcus epidermidis 949_S8	949_S8	unknown (nearest 536)	CGE	1	5	1	2	2	1	4	SAMN03339004	PRJNA274731	GCA_000934225.1	Ch	2.33987	32	chromosome:CP010942.1	2221	2119	24.02.15	05.08.15

[1]Multilocus sequence typing (MLST) with tool from Center for Genomic Epidemiology (CGE) or from indicated references.

[2]Complete Genome (CG), chromosome (Ch)

Supplementary Table 3: Summary of the variants found in the ST378 clinical isolates

	Position in RP62A	Type	Reference	Allele	Length	Gene name/function	AA change	Isolates	Cluster
1	90580	SNP	C	T	1	Multimodular transpeptidase-transglycosylase/ Penicillin-binding protein 1A/1B (PBP1)	synonymous	1A, 1B, 1C, 1D, 1E, 1F, 16J, 16N	
2	315658	SNP	G	A	1	GraR, two-component response regulator	Gly59Arg	1A, 1B, 1C, 1D, 1E, 1F,16J, 16N	
3	348486	SNP	C	A	1	Sodium/di- and tricarboxylate cotransporter	Ser161stop	1A, 1B, 1C, 1D, 1E, 1F,16J, 16N	Group I
4	990611..990612	Del	CG	-	2	Cold-shock protein CspA	Gly57fs	1A, 1B, 1C, 1D, 1E, 1F,16J, 16N	
5	1735899	SNP	G	T	1	RodA, rod shape-determining protein / FtsW, cell division protein	Gly161Val	1A, 1B, 1C, 1D, 1E, 1F,16J, 16N	
6	1864486	SNP	G	A	1	PbuG, Hypoxanthine/guanine permease	Ser27Leu	1A, 1B, 1C, 1D, 1E, 1F,16J, 16N	
7	188678	SNP	G	T	1	RpoB, DNA-directed RNA polymerase beta subunit	Arg917Leu	1A, 1B, 1C, 1D, 1E, 1F, 16N	
8	85098	SNP	T	C	1	Sle1, Autolysin, N-acetylmuramoyl-L-alanine amidase	Val67Ala	1A	
9	398168	SNP	C	T	1	NrdI, Ribonucleotide reduction protein	Arg14stop	1A	
10	1239544	SNP	C	T	1	SecDF, protein translocase subunit	Ser621Asn	1A	
11	2372735	SNP	G	C	1	Bicyclomycin resistance protein TcaB/ Major myo-inositol transporter IolT	Pro64Ala	1A	
12	169028..169048	Del	TTGTGCAT GTGCCAA CACACG	-	21	ClpC, ATP-dependent Clp protease ATP-binding subunit	Arg12_Gln18del	1B, 1E, 16N	
13	398462..398468	Del	GAATTTA	-	7	NrdI, Ribonucleotide reduction protein	Glu112fs	1B, 1E, 16N	★
14	1296398	SNP	C	G	1	isocitrate dehydrogenase	Val356Leu	1B, 1E, 16N	
15	10897	SNP	A	G	1	Putative metal chaperone, involved in Zn homeostasis, GTPase of COG0523 family	synonymous	1E	
16	92614	SNP	T	C	1	membrane protein, putative	synonymous	1E	
17	435659..435660	SNP	G	C	1	SufB, Fe-S cluster assembly protein	Ala129Gly	1E	
18	1211190	SNP	C	G	1	peptidase, U32 family large subunit [C1]	Glu90Gln	1E	
19	1182887	SNP	T	C	1	PrmA, ribosomal protein L11 methyltransferase	Asp107Gly	1B, 16N	
20	1923243	SNP	G	A	1	Sialic acid utilization regulator, RpiR family / MurR/RpiR family transcriptional regulator	Val84Ile	1B, 16N	■
21	2589790	SNP	A	G	1	Two-component sensor kinase WalK	Met428Thr	1B, 16N	
22	9376	SNP	A	G	1	hypothetical protein	synonymous	16N	
23	279591	SNP	C	T	1	SarA, Staphylococcal accessory regulator A	Ala70Thr	16N	
24	1312426..1312429	Del	TTTG	-	4	3'-to-5' oligoribonuclease A	Pro46fs	16N	
25	1319725	SNP	T	C	1	acetate kinase	Thr239Ala	16N	
26	1970731..1970742	Del	GAAGCAC CAGGT	-	12	Mqo, malate:quinone oxidoreductase	Ser431_Pro432_ Gly433_Ala434del	16N	
27	1912086	SNP	G	C	1	Salicylate hydroxylase	Ala290Gly	1C, 1D, 1F	●
28	294063..294064	Del	TA	-	2	Manganese ABC transporter, inner membrane permease protein SitD	Tyr243fs	1C	
29	398288	SNP	C	T	1	NrdI, Ribonucleotide reduction protein	Gln54stop	1C	
30	1219569	SNP	A	G	1	YrrC, RecD-like DNA helicase, deoxyribonuclease	Ile122Thr	1C	
31	40832	SNP	A	T	1	RpoC, DNA-directed RNA polymerase beta' subunit	synonymous	1D, 1F	
32	190909	SNP	G	T	1	RpoC, DNA-directed RNA polymerase beta' subunit	Gly433Val	1D, 1F	▲
33	1099372	SNP	T	C	1	RibU, Riboflavin transporter	Met1Val	1D, 1F	

Blue shading indicates isolate group I and red shading isolate group II. The purple highlighted genes are likely to be under a general selection pressure within the host, as they are mutated in different positions and across many branches of the phylogenetic tree. The symbols in last column are a guide to visualize the mutations characteristic to a specific cluster, they are shown on the corresponding tree branches in Figure 2B. Deletion (Del), Insertion (Ins), single nucleotide polymorphism (SNP).

Supplementary Table 3 (Continuation)

	Position in RP62A	Type	Reference	Allele	Length	Gene name/function	AA change	Isolates	Cluster
34	489412	SNP	A	G	1	Iron-sulfur cluster assembly scaffold protein NifU	Lys134Glu	1D	
35	124653	SNP	T	C	1		Phe33Ser	1F	
36	1101687	SNP	A	C	1	SrrB, respiratory response protein	Ile13Ser	1F	
37	186338	SNP	A	C	1	RpoB, DNA-directed RNA polymerase beta subunit	Gln137Pro	16J	
38	331340^331341	Ins	-	AG	2	Predicted RNA-binding protein, associated with RNAse of E/G family	Val15fs	16J	
39	524546	Del	T	-	1	general stress protein 13 (contains ribosomal protein S1 (RPS1) domain)	Thr31fs	16J	
40	1970921	SNP	G	A	1	Mqo, malate:quinone oxidoreductase	Arg375Trp	16J	
41	1503567	SNP	T	A	1	PutP, Proline/sodium symporter	Leu353stop	1A, 1B, 1C, 1D, 1E, 1F,16J, 16N, 14G,16L,16O	
42	243329	SNP	G	T	1	Pta, Phosphate acetyltransferase	Asp245Tyr	14G, 16H,16I, 16K, 16L, 16M,16O	
43	743713	SNP	G	T	1	penicillin-binding protein 1/Cell division protein FtsI	Val605Leu	14G, 16H,16I, 16K, 16L, 16M,16O	
44	1724485	SNP	G	T	1	RsbU, sigma factor B regulator	Thr325Asn	14G, 16H,16I, 16K, 16L, 16M,16O	Group II
45	1788877	SNP	C	A	1	RNA-binding protein, Conserved Protein Domain Family EVE RNA binding	Trp17Cys	14G, 16H,16I, 16K, 16L, 16M,16O	
46	1007576..1007577	Del	AA	-	2	ArlR, two-component response regulator	Leu51fs	14G, 16H,16I, 16K, 16L, 16M,16O	
47	1971641	SNP	G	A	1	Mqo, malate:quinone oxidoreductase	Arg132stop	14G, 16H,16I, 16K, 16L, 16M,16O	
48	187392^187393	Ins	-	GGGCCT	6	RpoB, DNA-directed RNA polymerase beta subunit	Gly491_Gly492 insProGly	14G, 16L,16O	
49	1327856	SNP	G	A	1	MsrC, Free methionine-(R)-sulfoxide reductase	Gly64Asp	14G, 16L,16O	★
50	2219210	SNP	G	T	1	Sat, sulfate adenylyltransferase	Pro348Gln	14G, 16L,16O	
51	2362000	SNP	C	T	1	pyruvate dehydrogenase subunit beta / branched-chain alpha-keto acid dehydrogenase E1	Glu201Lys	14G, 16L,16O	
52	948144^948145	Ins	-	TTGAAC	6	MprF virulence factor, phosphatidylglycerol lysyltransferase	Glu791_His792 insLeuGlu	16L	
53	84065	SNP	A	T	1	GdpP, Phosphoesterase, DHH family protein	synonymous	16L,16H, 16I, 16K	
54	827107^827108	Ins	-	A	1	GTP-sensing transcriptional pleiotropic repressor CodY	Glu254fs	16H, 16I, 16K, 16M	
55	879115	SNP	G	C	1	glycerol-3-phosphate responsive antiterminator	Gly129Ala	16H, 16I, 16K, 16M	■
56	1182494	SNP	T	G	1	PrmA, ribosomal protein L11 methyltransferase	Glu238Ala	16H, 16I, 16K, 16M	
57	187403	SNP	G	T	1	RpoB, DNA-directed RNA polymerase beta subunit	Gly492Val	16I	
58	579464	Del	C	-	1	RelQ, (p)ppGpp synthetase	Phe40fs	16I	
59	1233442	SNP	C	A	1	RSH, (p)ppGpp synthase/hydrolase	Leu533Phe	16H, 16K, 16M	
60	187344^187345	Ins	-	GCT	3	RpoB, DNA-directed RNA polymerase beta subunit	Ala473_Asn474 insAla	16H, 16K, 16M	●
61	1968672	SNP	A	G	1	HssR, heme response regulator	Thr95Ala	16H, 16K, 16M	
62	1560546	Del	A	-	1	AgrA, accessory gene regulator protein A	Glu42fs	16H	
63	2463079	SNP	C	A	1	Hypothetical serine protease	Arg78Ile	16H	
64	1561074	SNP	G	C	1	AgrA, accessory gene regulator protein A	Arg218Pro	16M	
65	2023218..2023223	Del	ATATGT	-	6	L-Cystine ABC transporter, periplasmic cystine-binding protein TcyA	Thr45_Tyr46del	16M	

References

1) Conlan, S. et al. (2012) ***Staphylococcus epidermidis* pan-genome sequence analysis reveals diversity of skin commensal and hospital infection-associated isolates,** Genome Biology. BioMed Central Ltd, 13(7), p. R64. doi: 10.1186/gb-2012-13-7-r64.

2) Lee, J. Y. H. et al. (2016) **Functional analysis of the first complete genome sequence of a multidrug resistant sequence type 2 *Staphylococcus epidermidis***, Microbial Genomics, 2(9), pp. 1–15. doi: 10.1099/mgen.0.000077.

3) Savijoki, K. et al. (2014) **Genomics and proteomics provide new insight into the commensal and pathogenic lifestyles of bovine- and human-associated *Staphylococcus epidermidis* strains**, Journal of Proteome Research, 13(8), pp. 3748–3762. doi: 10.1021/pr500322d.
