# Supplementary material for: In-host evolution of Staphylococcus epidermidis in a pacemaker-associated endocarditis resulting in increased antibiotic tolerance
Source: Nat Commun. 2019 Mar 8;10:1149. doi: 10.1038/s41467-019-09053-9 (PMC6408453; doi:10.1038/s41467-019-09053-9)
Supplement: Supplementary file 3 — Description of Additional Supplementary Files [file 41467_2019_9053_MOESM3_ESM.docx]

**Description of Supplementary Files**

**File Name:** Supplementary Data 1

**Description:** SNPs and InDels found in all clinical ST378 S. epidermidis isolates compared to the reference S. epidermidis strain RP62A. The 13'658 identical variants found in all isolates when compared to the reference. These variants were excluded for the comparative purposes of our analysis. Only the variants unique to one isolate or a cluster of isolates are reported.
